# Validation of the Clinical COPD questionnaire in Italian language

**DOI:** 10.1186/1477-7525-3-9

**Published:** 2005-02-07

**Authors:** Salvatore Damato, Chiara Bonatti, Vinicio Frigo, Silvana Pappagallo, Rita Raccanelli, Claudio Rampoldi, Francesco Rodi

**Affiliations:** 1 Department of Clinical Medicine and Prevention, University of Milano-Bicocca, Via Cadore 48, 20052 Monza (MI), Italy; 2 Division of Pulmonary Rehabilitation, Ospedale Trabattoni-Ronzoni, Via Verdi, 2 20048 Seregno (MI), Italy

## Abstract

**Background:**

The development and validation study of the Clinical Chronic Obstructive Disease (COPD) Questionnaire (CCQ) has recently been published in this journal. The CCQ is the first questionnaire that incorporates both clinician and patient guideline goals in the clinical control evaluation of patients with COPD in general clinical practice. The aim of this study is the validation of the CCQ questionnaire in Italian, in specific pulmonary disease clinical practice.

**Methods:**

Validity was tested on a population of healthy subjects and patients with COPD, using the Italian validated version of the Short Form Health Survey (SF-36) and guideline recommended routine measurement in COPD patients (FEV_1_, FVC, BMI and functional dyspnoea). Test-retest reliability was tested by re-administering the CCQ after 2 weeks. Responsiveness was tested by re-administering the CCQ after three weeks of hospital pulmonary rehabilitation. Distance walked and Borg breathlessness rating were measured at the end of the six-minute walking test (6 MWT), before and after rehabilitation.

**Results:**

Cross-sectional data were collected from 175 subjects (55 healthy; 40 mild-moderate, 50 severe and 25 very severe COPD). Cronbach's alpha was high (0.89). The CCQ scores in patients were significantly worse than in healthy subjects. The CCQ total score in patients with COPD was significantly worse in those with BMI < = 21. Significant correlations were found between the CCQ total score and domains of the SF-36 (rho = -0.43 to rho = -0.72). The correlation between the CCQ and FEV1 % predicted was rho = -0.57. The correlation between the CCQ and MRC was rho = 0.63. Test-retest reliability was determined in 112 subjects over a period of two weeks (Intra Class Coefficient = 0.99). Forty-six patients with COPD showed significant improvement in CCQ scores, distance-walked and Borg breathlessness rating after 3 weeks of pulmonary rehabilitation, indicating CCQ responsiveness.

**Conclusions:**

The CCQ is self-administered and has been specially developed to measure clinical control in patients with COPD. Data support its validity, reliability and responsiveness in Italian and in specific pulmonary disease clinical practice.

## Background

The American Thoracic Society (ATS) and European Respiratory Society (ERS) have jointly proposed standards [1] for the diagnosis, treatment and spirometric classification of patients with chronic obstructive pulmonary disease (COPD). According to the GOLD (Global Obstructive Lung Disease) guideline [2], the goals of clinical control in patients with COPD include health-related quality of life goals (improved exercise tolerance and emotional function) and clinical goals (prevention of disease progression and minimization of symptoms).

The Clinical COPD questionnaire (CCQ) [3] is the first practical clinical instrument to be used for routine evaluation of clinical control (symptom, functional state and mental state) concerning patients with COPD, in general practice. The development and validation study has been published in this journal and data were collected from 119 subjects. The aim of the present study is the validation of the CCQ in Italian in specific pulmonary disease clinical practice. In this practice, the ATS/ERS [1] recommended routine measurements, in all patients with COPD, are the following: forced expiratory volume in one second (FEV_1_) and forced vital capacity (FVC), body mass index (BMI) and functional dyspnoea (Medical Research Council – MRC).

## Methods

### Subjects

Healthy subjects were selected in social meeting places. Subjects were asked, individually, to answer a simple questionnaire after the study had been explained to them. Only subjects over 40 years of age were interviewed. We excluded subjects with any disease symptoms, or any limitation in daily activities for any reason, or who mentioned suffering from disabling chronic diseases (COPD, asthma, arthritis, angina or heart insufficiency). All subjects gave their informed written consent for baseline spirometry and questionnaires administration, as approved by the local Medical Ethics Committee. We enrolled 55 subjects, 52 non-smokers and 3 ex-smokers. Subject data are shown in Table 1.

**Table 1:** Characteristics and results of the study population in subgroups

	**Healthy subjects**	**Mild-moderate COPD-class I-II**	**Severe COPD-class III**	**Very severe COPD-class IV**
N	55	40	55	25
Males (%)	62.0	85.0	63.6	72.0
Age (yr)	70^abcd ^(41–82)	72^abcd ^(58–84)	71^abcd ^(41–86)	71^abcd ^(42–86)
LTOT (%)	0.0	0.0	32.7	72.0
HMV (%)	0.0	0.0	7.2	8.0
BMI (kg/m^2^)	25.7^abcd ^(18.0–30.0)	26.7^abcd ^(18.6–37.8)	25.1^abcd ^(16.4–36.4)	26.6^abcd ^(16.2–34.6)
FEV_1_/FVC (%)	79.2 (70.4–94.5)	59.7 (40.4–68.2)	44.2 (27.9–66.2)	35.1 (21.1–57.3)
FEV_1 _(% predicted)	108.0 (69–132)	69.5 (51.4–117.1)	40.7 (30.2–49.8)	26.4 (16.4–29.7)
MRC functional dyspnoea	0.6 ± 3.4 (0–1)	1.1 ± 0.8 (0–2)	1.6 ± 0.7 (0–4)	2.3 ± 0.9 (0–4)
CCQ symptom	0.5 (0.0–4.0)	1.3^b ^(0.0–4.0)	1.5^b ^(0.3–5.8)	2.5 (0.3–3.8)
CCQ functional state	0.5^a ^(0.0–5.3)	1.0^a ^(0.0–3.5)	1.5 (0.0–5.3)	3.0 (0.3–5.0)
CCQ mental state	0.0 (0.0–4.5)	0.0 (0.0–5.0)	1.0^c ^(0.0–6.0)	1.5^c ^(0.0–6.0)
CCQ total	0.4 (0.0–3.8)	0.9 (0.0–3.5)	1.4 (0.3–5.2)	2.6 (0.4–4.3)

CCQ = Clinical COPD Questionnaire; range 0–6; 0 indicating best possible control and 6 indicating worst clinical control. LTOT = long term oxygen therapy. HMV = home assisted mechanical ventilation during night. BMI = body mass index. FVC = forced vital capacity. FEV_1 _= forced expired volume in one second. MRC = Medical Research Council. Healthy: normal spirometry, no chronic symptoms (cough, sputum production and/or dyspnoea). COPD classification by post-bronchodilator spirometry according to GOLD guidelines: mild-moderate FEV_1_/FVC <= 0.70 and FEV_1 _>= 50% predicted, severe FEV_1_/FVC <= 0.70 and FEV_1 _>= 30% predicted, very-severe FEV_1_/FVC <= 0.70 and FEV_1 _< 30% predicted. Medians not sharing a common superscript (a,b,c,d) are significantly different at p < 0.05 after Mann-Wittney U test.

MRC data are reported as mean value with standard deviation and range.

Patients with COPD were consecutively enrolled in the outpatient section of our Division during medical consultation. According to the guidelines [1,2], COPD was defined by the presence of chronic cough, sputum production and/or dyspnoea. Patients with airways obstruction (FEV_1_/FVC <= 0.70) were classified as mild (FEV_1 _post-bronchodilator (pb) >= 80% predicted), moderate (FEV_1 _pb >= 50% predicted), severe (FEV_1 _pb >= 30% predicted) and very severe (FEV_1 _pb < 30% predicted). We excluded COPD patients with: a) significant improvement of FEV_1 _pb (>= 15 % and/or 200 ml) compared with baseline, b) disease exacerbation in the previous four weeks, c) asthma, chronic heart failure, obstructive sleep apnoea syndrome, cancer or other disabling diseases except COPD. We enrolled 120 patients (77 ex-smokers, 19 smokers). In 1999, the local health service authority approved the standard evaluation procedures used in our outpatient clinic for patients with COPD. The patients' data are shown in Table 1.

Forty-six patients with COPD (exclusion criteria as mentioned above) were enrolled in a continuous pulmonary rehabilitation program, 31 males, 30 ex-smokers, 6 smokers, 13 in long-term oxygen therapy (LTOT), 2 in home-assisted mechanical ventilation during the night (HMV), median age 72 (range 41–83), median FEV_1 _pb 46 % predicted (range 18–68). In 1999, the local health service authority approved our continuous pulmonary rehab program for patients with COPD.

### Cross sectional validity

The CCQ was administered to all subjects. They were instructed to recall their experiences during the previous week. The CCQ is self-administered and contains only 10 items, subdivided into three domains: symptom (item 1–2–5–6), functional state (item 7–8–9–10) and mental state (item 3–4). Subjects responded to each question using a 7 point scale from 0 = asymptomatic or no-limitation, to 6 = extremely symptomatic or totally limited. The overall clinical COPD control score and the score of the three domains was calculated by adding all the scores together and dividing the sum by the number of questions. The Italian translation of the copyrighted questionnaire and permission for use was obtained from T. van der Molen [3] in February 2004 by one of the team (SD).

Lung function (FEV1 and FVC) was measured according to ERS guidelines [4] using a portable turbine spirometer (Pony, Cosmed, Italy) in base condition (all subjects) and 20 minutes after metered inhalation of 200 mcg of salbutamol (COPD patients only).

The copyrighted Italian validated version [5] of the 36-item Short Form Health Survey (SF-36) [6], a generic health-related quality of life questionnaire, was administered to 120 patients with COPD and 55 healthy subjects. The validated Italian version of SF-36 and permission for use was obtained from GlaxoSmithKline in June 2002 by one of the team (SP). Functional dyspnoea was assessed in all subjects using the Medical Research Council (MRC) scale as proposed by ATS/ERS guidelines [1]: 0 = not subject to breathlessness except with strenuous exercise, 1 = subject to shortness of breath when hurrying or walking up a gradually sloping hill, 2 = walks slower than people of the same age due to breathlessness or has to stop for breath when walking at a normal pace on a level, 3 = stops for breath after walking about 100 m or after a few minutes on a level, 4 = too breathless to leave the house or breathless when dressing or undressing. BMI was calculated by dividing weight (in kg) over height (in m^2^), for all subjects.

### Longitudinal validity

The CCQ was re-administered after 2 weeks (where there was no variation of the previous therapy or introduction of new therapy) in 112 subjects (53 healthy and 59 patients with COPD), 75 males, median age 71 years (range 41–84), median FEV_1 _60 % predicted (range 19–117). We tested the CCQ responsiveness in patients with COPD undergoing continuous pulmonary rehabilitation. Patients were treated in four successive groups in our hospital following a standard three-week protocol. According to guidelines [7], the program was individually tailored and designed to optimize physical and social performance and autonomy, and to be integrated into overall patient treatment. It was a mix of physical retraining, thoracic and general physiotherapy, education, self-monitoring. At the end of the three-week hospitalization period, patients received: a) their individual continuous pulmonary rehabilitation home program together with optimized pharmacological therapy, b) the next three-month appointment for the outpatient evaluation visit, c) the next six-month appointment for successive inpatient three-week pulmonary rehabilitation. The CCQ was administered, on admission and on discharge from hospital, to 46 COPD patients. Patients were submitted to a 6-minute walking test (6 MWT) on hospital admission and discharge, according to guidelines [8]. In each occasion, we measured distance-walked and breathlessness at the end of 6 MWT, using the standard Borg rating scale [9]. This is a category scale in which simple verbal expressions, that describe increasing degrees of breathlessness in exercise, are linked to numbers (range from 0 = nothing at all to 10 = maximal). The CCQ was also administered after two more weeks during home-based comprehensive treatment. All patients gave their informed written consent for re-administration of CCQ, at home or in the outpatient section.

### Statistical Analysis

We applied the same analysis undertaken in the original English validation study [3], taking for granted the same a priori assumptions. Data analysis was performed using SPSS version 12.0 (SPSS Inc, USA). Data are expressed as median (range) unless otherwise stated. CCQ internal consistency was evaluated by calculating Cronbach's alpha coefficient (for the three domains and the total). Non-parametrical testing (Mann-Whitney U test) was used to determine the discriminant validity of the CCQ to differentiate between healthy subjects and COPD patients with different degrees of airways obstruction (mild, moderate, severe, very severe). Spearman's rank correlations were used to examine convergent and divergent validity. Test-retest reliability analysis was done by calculating the Intra-class Correlation Coefficient (ICC). Responsiveness was tested using Wilcoxon U test. A value of p < 0.05 was considered as statistically significant.

## Results

### Score distributions

The distributions for all domains and the overall scores were skewed. In the population study, 12 subjects (7%) scored optimally (= 0) in the total score, whereas 87 subjects (50%) scored optimally in the mental state domain. In the COPD group (120 subjects), 3% of the patients scored optimally in the total score, whereas 35% scored optimally in the mental state.

### Internal consistency

Cronbach's alpha was 0.89 for the total score. Internal consistencies of symptom, functional state and mental state were 0.71, 0.88 and 0.80, respectively.

### Discriminant validity

Healthy subjects had significantly lower (better) CCQ score only in the symptom domain (p = 0,05) compared with the small number (6 subjects) of patients with mild COPD (FEV_1 _pb >= 80% predicted). At the same time, this small group did not differ significantly (all CCQ scores) from the group (34 patients) with moderate COPD (FEV_1 _pb < 80% and >= 50% predicted). For this reason, Table 1 shows CCQ scores in subgroups of healthy, mild-moderate COPD, severe COPD, and very severe COPD subjects.

Healthy subjects had significantly lower CCQ scores than patients with mild-moderate COPD with respect to total score (p = 0.001), symptom domain (p = 0.000), mental state domain (p = 0.005), except functional state domain. Patients with mild-moderate COPD had better CCQ values compared with patients with severe COPD, with respect to total score (p = 0.041), functional state (p = 0.017), mental state (p = 0.037), except symptom domain. Patients with severe COPD had lower CCQ scores than patients with very severe COPD, with respect to total score (p = 0.007), functional state domain (p = 0.003), symptom domain (p = 0.032) except mental state domain.

The healthy subjects group had a significantly (p = 0.003) lower (better) MRC score than patients with mild-moderate COPD. Patients with severe COPD had a significantly (p = 0.003) higher (worse) MRC score than patients with mild-moderate COPD. Patients with very severe COPD had a significantly (p = 0.000) worse MRC score compared with those with severe COPD (Table 1).

We did not find (Table 1) any significant difference in BMI between healthy and diseased subjects and among patients with increasing airways obstruction. In agreement with Celli BR et al. [10], we considered 21 as a cut-off BMI value for COPD patients' clinical control. Table 2 shows the data of 120 patients with COPD subdivided into three different classes: subjects having BMI <= 21 (low-range), BMI <= 28 (acceptable-range) and BMI > 28 (high-range). In these three groups, no significant difference was found for FEV_1 _pb % predicted, age and MRC score. CCQ scores were higher in both low and high BMI groups, with respect to the acceptable BMI range group. CCQ scores did not indicate any significant difference between acceptable-range and high-range groups, except in the CCQ mental state domain (p = 0.02). On the other hand, there was a statistically significant difference between low-range BMI and acceptable-range BMI groups for CCQ total (p = 0.01), CCQ symptom (p = 0.01), CCQ mental state (p = 0.04) except CCQ functional state.

**Table 2:** Characteristics and results of 120 patients in subgroups by BMI

	**BMI <= 21**	**BMI <= 28**	**BMI >28**
N	15	66	39
Males (%)	60.0	78.8	66.7
Age (yr)	71^abc ^(42–86)	72^abc ^(41–86)	71^abc ^(50–82)
BMI (kg/m^2^)	19.7 (16.2–20.8)	25.0 (21.3–27.8)	29.9 (28.0–37.8)
FEV_1_/FVC (%)	39.0^abc ^(21.1–64.8)	49.3^abc ^(23.6–68.0)	50.3^abc ^(27.5–68.2)
FEV_1 _(% predicted)	43.5^abc ^(18.9–68.1)	44.5^abc ^(19.5–117.1)	40.8^abc ^(16.4–85.9)
MRC functional dyspnoea	1.8 ± 1.4^abc ^(1–4)	1.4 ± 0.4^abc ^(0–4)	1.7 ± 0.9^abc ^(0–4)
CCQ symptom	2.5 (0.3–5.8)	1.3^a ^(0.0–4.0)	1.5^a ^(0.0–5.0)
CCQ functional state	2.3^a ^(0.0–5.0)	1.3^ab ^(0.0–5.3)	1.9^ab ^(0.0–4.5)
CCQ mental state	2.0 (0.0–5.5)	0.5 (0.0–6.0)	1.5 (0.0–6.0)
CCQ total	2.2 (0.4–5.2)	1.2^a ^(0.0–4.3)	1.7^a ^(0.0–4.6)

BMI = body mass index. FVC = forced vital capacity. FEV_1 _= forced expired volume in one second. CCQ = Clinical COPD Questionnaire. MRC = Medical Research Council. Medians not sharing a common superscript (a,b,c) are significant different at p < 0.05 after Mann-Wittney U test.

### Convergent and divergent validity

The CCQ score showed significant correlations with all SF-36 components except the pain component(Table 3).

**Table 3:** Correlations between CCQ, SF-36, FEV_1 _and functional dyspnoea

	**CCQ Symptom**	**CCQ Functional state**	**CCQ Mental state**	**CCQ Total**
SF-36 Physical functioning	-0.51**	-0.78**	-0.45**	-0.72**
SF-36 Social functioning	-0.36*	-0.40**	-0.40**	-0.43**
SF-36 Role physical	-0.34*	-0.38**	-0.43**	-0.43**
SF-36 Role emotional	-0.31*	-0.30*	-0.39*	-0.36*
SF-36 Mental health	-0.35*	-0.47**	-0.54**	-0.48**
SF-36 Vitality	-0.47**	-0.58**	-0.44**	-0.57**
SF-36 Pain	-0.15	-0.23	-0.05	-0.20
SF-36 Health perceptions	-0.56**	-0.58**	-0.49**	-0.64**
MRC functional dyspnoea	+0.52**	+0.64**	+0.44**	+0.63**
FEV_1 _(% predicted)	-0.51**	-0.50**	-0.51**	-0.57**

SF-36 = Medical Outcome Survey Form-36 (higher score indicates better health status); FEV_1 _= forced expired volume in one second; MRC = Medical Research Council functional dyspnoea; *p < 0.05; **p < 0.01, Spearman's rank correlation.

**Figure 1:**
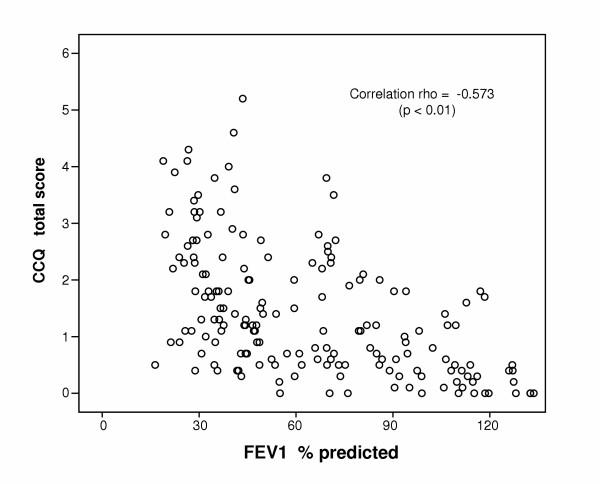
**Correlation between CCQ and FEV1 %predicted in 175 subjects. **CCQ = Clinical COPD Questionnaire. FEV_1 _= forced expired volume in one second.

The CCQ scores and the FEV_1 _% predicted values correlated significantly with respect to the whole population, the highest correlation (Figure 1) being that of CCQ total score (rho = -0.57; p < 0.01). The correlation (rho = -0.41) was highly significant (p < 0.01) even if only the group of 120 COPD patients is considered.

The functional dyspnoea MRC score correlated strongly with CCQ total (rho = 0.63), functional state (rho = 0,64), symptom (rho = 0.52) and mental state (rho = 0.44).

No significant correlation was found between BMI and all the CCQ scores.

### Test-Retest Reliability and Responsiveness

The intra-class correlation coefficient was 0.99 for the overall CCQ score.

In table 4 we see the results concerning responsiveness to change of the CCQ, as tested in 46 COPD patients undergoing pulmonary rehabilitation. The group's CCQ scores significantly (p = 0.000) improved after three weeks of pulmonary rehabilitation in hospital. A statistically significant (p = 0.000) improvement was found also for walked distance and Borg breathlessness rating at the end of 6 MWT. At the same time, no significant change was found for the FEV_1 _pb %. After two successive weeks of individualized home rehabilitation there was a worsening of CCQ scores compared with the scores when hospital discharge took place. Nevertheless, CCQ scores were still significantly better than in baseline condition (hospital admission) for total (p = 0.01), functional state (p = 0.01), symptom (p = 0.02) and mental state (p = 0.03).

**Table 4:** Changes of CCQ scores in 46 patients submitted to pulmonary rehabilitation

	**Baseline**	**HospitalR**	**HomeR**
CCQ functional state	2.2 (0.5–5.0)	1.5 (0.0–4.7)*	1.7 (0.0–4.7)*
CCQ symptom	1.8 (0.2–4.7)	1.3 (0.0–3.5)*	1.7 (0.0–4.2)*
CCQ mental state	2.0 (0.5–4.0)	1.0 (0.0–3.5)*	1.5 (0.0–4.5)*
CCQ total	2.0 (0.0–3.9)	1.3 (0.0–3.8)*	1.7 (0.0–4.0)*
Distance_walked (m)	264 (104–380)	306 (156–459)	-----
Borg end-walking	2 (1–9)	1 (0–8)	-----

CCQ = Clinical COPD Questionnaire, HospitalR = end of hospital pulmonary rehabilitation, HomeR = during home rehabilitation. Borg = breathlessness rating scale. *p < 0.05 after Wilcoxon U test (HospitalR or HomeR versus Baseline)

## Discussion

The validated Italian version of SF-36 was used as an instrument to measure the convergent validity of the clinical COPD questionnaire. Moderate to high correlations were found in the present study supporting the convergent validity in the Italian version, reflecting the original English development and validation study [3]. FEV_1 _was used to measure divergent validity with the same a priori assumption behind the original English version (range from -0.20 to -0.4). The correlation was stronger than expected also in the Italian version, concerning the whole population study and the COPD population alone.

In addition to the FEV_1_, also MRC functional dyspnoea has proved to be useful in predicting outcomes in patients with COPD, thus MRC functional dyspnoea measurement is recommended in the routine handling and evaluation of these patients [1]. In the present study, both MRC score and CCQ scores values were able to discriminate healthy subjects and COPD patients with different degree of airways obstruction (from mild-moderate to very severe). We had the opportunity of testing the correlation between CCQ scores and MRC score and it was statistically highly significant.

We found no significant difference, as far as the CCQ functional state is concerned, between healthy subjects and mild-moderate patients with COPD. This reflects COPD guidelines [1], which state that restrictions in daily living activities only become significantly apparent once the FEV_1 _falls below 50% predicted, i.e., as a result of the transition from mild-moderate to severe airways obstruction in patients with COPD.

BMI calculation has also proved to be useful in the routine handling of patients with COPD [1]. In the present study, BMI does not differ significantly between healthy and diseased subjects and among groups of COPD patients with different degrees of airways obstruction. According to Celli BR et al. [10], BMI <= 21 is associated with poor prognosis in patients with COPD. Therefore, this condition can be considered an indication of less than optimal clinical control in patients with COPD. In our study, CCQ scores were able to discriminate the patients with COPD and BMI <= 21 in the COPD population. The relation between BMI and CCQ scores in our COPD population is non-linear, since scores tend to be worse with both decreasing BMI values below 22 and increasing values above 28. This trend, which is statistically significant only in the low BMI range, explains the non-significant overall correlation that was found between BMI values and CCQ scores in patients with COPD. Only the CCQ mental state score is significantly worse in the overweight group, compared with the acceptable BMI range group. This would suggest the presence in these subjects of emotional problems, possibly related also to overfeeding.

We have been able, by means of the CCQ scores, to detect significant changes in response to the inpatient portion of a comprehensive and continuous standard pulmonary rehabilitation program for patients with COPD. Disease control improvement is also documented with independent outcome measurements of variables at the end of 6 MWT. It is a well-known fact that improvements in clinical disease control and health status occur with pulmonary rehabilitation, despite a minimal effect on pulmonary function measurement, i.e., FEV1 % predicted [1,7,11]. The present study wishes to validate the Italian language version of the CCQ questionnaire; it does not intend to validate the optimal duration of a time-limited pulmonary rehab program. The GOLD guidelines [2] state that there is type B scientific evidence for two-month duration of a time-limited pulmonary rehab program in patients with COPD. In our clinical practice, we have never succeeded in obtaining the compliance of patients with stable COPD over such a long pulmonary rehab hospitalization period.

The comparison of our data with the results presented in the original CCQ article [3] show similar CCQ scores as far as the healthy subjects group is concerned (total score < 1). A separate comparison for severe and very severe groups of patients with COPD was impossible since the original CCQ article [3] presents data in these patients, grouped according to the classification criteria available in January 2003. Indeed, the classification of patients into different groups has been changed from one based upon the relation between airways obstruction and clinical features (respiratory failure or clinical signs of heart failure) [12] into another based upon airways obstruction alone [1,2]. However, we excluded from the study the patients with signs of heart failure or acute respiratory failure.

In our study, a separate comparison between mild and moderate COPD was impossible, since patients with mild COPD seldom refer to our specialized outpatient clinic. Furthermore, in our clinical setting, we have been unable to find any subject presenting symptoms of COPD in the absence of airways obstruction (subjects who risk developing COPD). We believe such patients are more typical in a general practice setting, as is the case in the original CCQ development and validation study [3].

## Conclusions

The clinical COPD questionnaire is the first to have been specifically developed and validated to measure clinical control in patients with COPD in general practice [3]. The validation of the questionnaire, in Italian and in specific pulmonary disease clinical practice, confirms strong discriminative properties, test-retest reliability and responsiveness. Furthermore, the CCQ scores are highly correlated with the usual functional dyspnoea MRC scale and are able to discriminate COPD patients with already known poor prognosis according to the critical BMI index.

## Authors' contributions

SD designed the study, analyzed the results, performed the statistical analysis and drafted the manuscript. SP participated to the study design and organization, collected and elaborated the SF-36 data and helped to draft the manuscript. CB-VF-CR-RR-FR participated in the organization of the study, in the individual subjects clinical selection and in the results discussion. All Authors read and approved the final manuscript.

## References

[B1] Celli BR, MacNee W, committee members (2004). Standards for the diagnosis and treatment of patients with COPD: a summary of the ATS/ERS position paper. Eur Respir J.

[B2] Fabbri LM, Hurd SS, for the GOLD Scientific Committee (2003). Global strategy for the diagnosis, management and prevention of COPD: 2003 update. Eur Respir J.

[B3] van der Molen T, Willemse BWM, Schokker S, ten Haken NHT, Postma DS, Juniper EF (2003). Development, validity and responsiveness of the Clinical COPD Questionnaire. Health Qual Life Outcomes.

[B4] Siafrakas NM, Vermeire P, Pride NB, Paoletti P, Gibson J, Howard P, Yernault JC, Decramer M, Higenbottam I, Postma DS, Rees J (1995). Optimal assessment and management of chronic obstructive pulmonary disesase (COPD): a consensus statement of the European Respiratory Society. Eur Respir J.

[B5] Apolone G, Mosconi P (1998). The Italian SF36 Health Survey: translation, validation and norming. J Clin Epidemiol.

[B6] Ware JJ, Sherbourne CD (1992). The MOS 36-item short form health survey (SF-36): I. Conceptual framework and item selection. Med Care.

[B7] (1999). Pulmonary rehabilitation: official statement of the American Thoracic Society. Am J Respir Crit Care Med.

[B8] (2002). ATS Committee on Proficiency Standards for Clinical Pulmonary Function Laboratories. ATS statement: guidelines for the six-minute walk test. Am J Respir Crit Care Med.

[B9] Borg G (1970). Perceived exertion as an indicator of somatic stress. Scand J Rehabil Med.

[B10] Celli BR, Cote CG, Marin JM, Casanova C, Montes de Oca M, Mendez RA, Pinto Plata V, Cabral HJ (2004). The body-mass index, airflow obstruction, dyspnoea, and exercise capacity index in chronic obstructive pulmonary disease. N Engl J Med.

[B11] Lacasse Y, Wong E, Guyatt GH, King D, Cook DJ, Goldstein RS (1996). Meta-Analysis of respiratory rehabilitation in chronic obstructive pulmonary disease. Lancet.

[B12] Pauwels RA, Buist AS, Calverley PM, Jenkins CR, Hurd SS (2001). The GOLD scientific Committee. Global strategy for the diagnosis, management, and prevention of chronic obstructive pulmonary disease. NHLBI/WHO Global Initiative for Chronic Obstructive Lung Disease (GOLD). Workshop summary. Am J Respir Crit Care Med.

